# Recombination between Clonal Lineages of the Asexual Fungus *Verticillium dahliae* Detected by Genotyping by Sequencing

**DOI:** 10.1371/journal.pone.0106740

**Published:** 2014-09-02

**Authors:** Michael G. Milgroom, María del Mar Jiménez-Gasco, Concepción Olivares García, Milton T. Drott, Rafael M. Jiménez-Díaz

**Affiliations:** 1 Department of Plant Pathology and Plant-Microbe Biology, Cornell University, Ithaca, New York, United States of America; 2 Department of Plant Pathology and Environmental Microbiology, The Pennsylvania State University, University Park, Pennsylvania, United States of America; 3 College of Agriculture and Forestry, University of Córdoba, and Institute for Sustainable Agriculture, CSIC, Campus de Excelencia Internacional Agroalimentario, ceiA3, Córdoba, Spain; Georg-August-University of Göttingen Institute of Microbiology & Genetics, Germany

## Abstract

Most asexual species of fungi have either lost sexuality recently, or they experience recombination by cryptic sexual reproduction. *Verticillium dahliae* is a plant-pathogenic, ascomycete fungus with no known sexual stage, even though related genera have well-described sexual reproduction. *V. dahliae* reproduces mitotically and its population structure is highly clonal. However, previously described discrepancies in phylogenetic relationships among clonal lineages may be explained more parsimoniously by recombination than mutation; therefore, we looked for evidence of recombination within and between clonal lineages. Genotyping by sequencing was performed on 141 *V. dahliae* isolates from diverse geographic and host origins, resulting in 26,748 single-nucleotide polymorphisms (SNPs). We found a strongly clonal population structure with the same lineages as described previously by vegetative compatibility groups (VCGs) and molecular markers. We detected 443 recombination events, evenly distributed throughout the genome. Most recombination events detected were between clonal lineages, with relatively few recombinant haplotypes detected within lineages. The only three isolates with mating type *MAT1-1* had recombinant SNP haplotypes; all other isolates had mating type *MAT1-2*. We found homologs of eight meiosis-specific genes in the *V. dahliae* genome, all with conserved or partially conserved protein domains. The extent of recombination and molecular signs of sex in (mating-type and meiosis-specific genes) suggest that *V. dahliae* clonal lineages arose by recombination, even though the current population structure is markedly clonal. Moreover, the detection of new lineages may be evidence that sexual reproduction has occurred recently and may potentially occur under some circumstances. We speculate that the current clonal population structure, despite the sexual origin of lineages, has arisen, in part, as a consequence of agriculture and selection for adaptation to agricultural cropping systems.

## Introduction

Asexual reproduction has evolved from sexually reproducing ancestors in almost all major groups of eukaryotes, except birds and mammals, and confers several short-term advantages [1]. In the long term, however, strict asexuality is considered an evolutionary dead end because of the accumulation of deleterious mutations, which would be purged by recombination in sexual organisms [2], [3], [4]. Thus, asexual organisms are almost always associated with either the recent loss of sexuality or the occurrence of some degree of undetected or cryptic sexual reproduction [5], [6], . Nonetheless, approximately one fifth of all described fungal species have no known sexual stages [8]. In ascomycete fungi, asexuality has evolved independently many times from sexual ancestors [6]. For many asexual fungi, the sexual stage is either unknown or has been observed only rarely in the laboratory, and therefore, inferences of recombination and cryptic sex have been made indirectly from population-genetic analyses [9], [10], [11], [12] and, more recently, from the analysis of genomes showing molecular signs of sex [5], [13], [14], [15], [16].

*Verticillium dahliae* is a plant-pathogenic fungus in the Sordariomycetes of the Ascomycota (www.mycobank.org). Although the Sordariomycetes are associated with well-described sexual reproductive stages, e.g., *Neurospora crassa*, *V. dahliae* appears to reproduce only by mitotically-produced spores and persistent soil-borne dormant structures (microsclerotia). Not surprisingly, population structure is highly clonal [17], [18], [19]. *V. dahliae* causes vascular wilt in a broad range of dicotyledonous host plants [20], [21], [22]. The broad host range and long-term survival of microsclerotia in soil makes this pathogen difficult to manage in agricultural systems. Moreover, *V. dahliae* is known to disperse by the movement of soil, irrigation water, and with seed and vegetative propagation [23]. Some clonal lineages have been dispersed across broad geographic distances [17] by agriculture.

*V. dahliae* has a clonal population structure with little or no evidence of recombination. Before the widespread availability of molecular markers, isolates of *V. dahliae* were classified into vegetative compatibility groups (VCGs). In ascomycetes, VCGs represent a multilocus genotype defined by the alleles at multiple heterokaryon incompatibility (*het*) loci; individuals with the same alleles at all *het* loci are vegetatively compatible and can form stable heterokaryons [24], [25]. In *V. dahliae*, three VCGs (VCGs 1, 2 and 4) are divided into subgroups based on the strength of complementation in heterokaryon compatibility assays, resulting in VCG subgroups 1A, 1B, 2A, 2B, 4A, and 4B [26], [27]. Genotyping with molecular markers has shown, with a few exceptions described in the next paragraph, that each VCG in *V. dahliae* represents a distinct clonal lineage [17], [18], [19], [28], [29]. In addition, nearly all isolates of *V. dahliae* have the same mating type (*MAT1-2*) [30], [31]; isolates with mating-type idiomorph *MAT1-1* are rare [19], [32]. In fungi, both mating-type idiomorphs are required for sexual reproduction [33], with few exceptions [34]. Therefore, the potential for sexual reproduction in *V. dahliae* appears to be low, assuming that mating can only occur between individuals of opposite mating type. Moreover, a considerable number of chromosome rearrangements has been observed among isolates of *V. dahliae*, which are thought to be involved in adaptation to different hosts [35]. The extent of rearrangements observed is likely to interfere with meiosis and reduce the probability of successful sexual reproduction between existing lineages [36], [37], [38].

**Figure 1:**
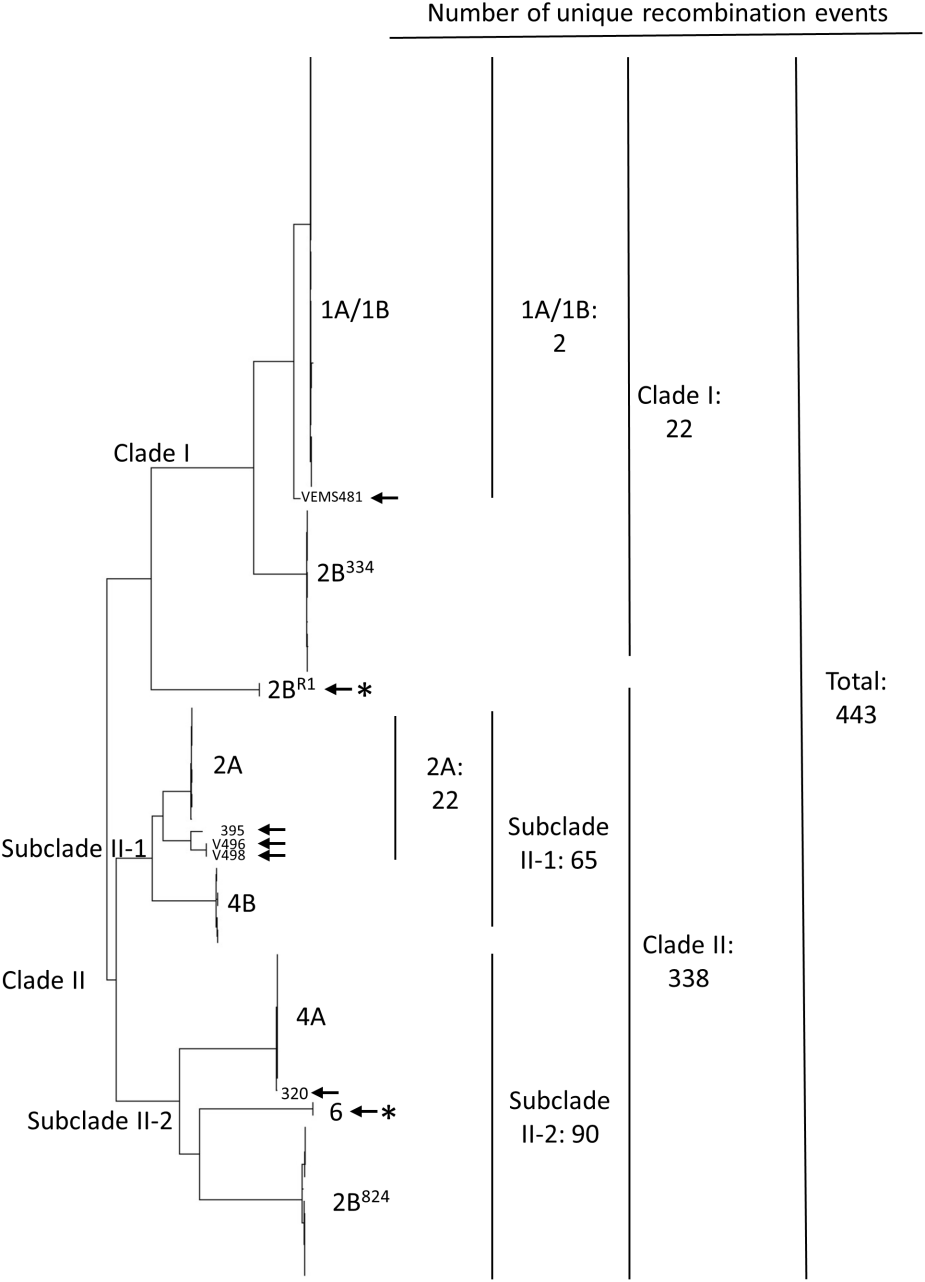
Relationships among clonal lineages and distribution of recombination events in *Verticillium dahliae*. The neighbor-joining tree was constructed in MEGA 5.10 [58] from 26,748 SNPs in 141 isolates of *V. dahliae*. Each clade or lineage had >99% bootstrap support out of the 1000 replicates. Clonal lineages (from top to bottom) 1A/1B, 2B^334^, 2A, 4B, 4A, 6 and 2B^824^ were named after their respective vegetative compatibility groups (VCGs) described previously. Lineage 2B^R1^ was not previously described. Recombinant lineages 2B^R1^ and 6 are indicated by arrows with an asterisk. Recombinant haplotypes are indicated by arrows, from isolates VEM481 (clade I), 395, V496 and V498 (clade II-1) and 320 (clade II-2). The number of unique recombination events associated with each hierarchy in the tree is shown on the right hand side (see also Table 3).

Despite the strong clonal structure and lack of evidence for sex in *V. dahliae*, some population-genetic evidence suggests that recombination may occur or has occurred in the past. First, high diversity of multilocus genotypes and the presence of all four dilocus genotypes (the four-gamete test) for some pairs of microsatellite loci were interpreted as evidence of recombination in *V. dahliae* [39]. A stronger argument for recombination derives from discrepancies in phylogenetic relationships among clonal lineages that could be explained more parsimoniously by recombination than mutation. The most obvious example is the polyphyletic nature of VCG2B, which comprises two distinct lineages, designated 2B^334^ and 2B^824^ [17], [18], [40], [41]. The probability of the same multilocus *het* genotype (which defines a VCG) arising independently in different lineages by mutation cannot be estimated for *V. dahliae* because the underlying genetics of VCGs and mutation rates at *het* loci are not known. In another discrepancy, subgroups of VCGs 2 and 4 (namely A and B) occur in distinct phylogenetic lineages defined by molecular markers [17], [18], [41]. VCG2A is more closely related to VCG4B than it is to either lineage of VCG2B; and VCG2B^824^ is more closely related to VCG4A than it is to VCG2A (or to VCG2B^334^) (Figure 1). This pattern is unlikely to arise by mutation if subgroups A and B within VCGs have common origins. Finally, additional incongruities have recently been shown in phylogenetic relationships inferred among lineages from nucleotide sequences of the intergenic spacer (IGS) region of the ribosomal RNA genes and by single-nucleotide polymorphisms (SNPs) from six polymorphic sequences [32], [41]. Collectively, these various types of evidence raise the question of whether some clonal lineages arose by recombination.

Analyses of fungal genomes have revealed molecular “signs of sex” [5] in supposedly asexual fungi. For example, functional mating-type genes were found in *Aspergillus* species [42], [43], [44], which previously had no known sexual stages. Arbuscular mycorrhizal fungi (AMF) in the Glomeromycota have long been thought to represent ancient asexual lineages [45], [46]. However, molecular signs of sex have recently been found that indicate they may have retained the ability to reproduce sexually. Population-genetic evidence, the presence of mating-type genes and the presence of genes that function in meiosis have recently been found in sequenced genomes of AMF species [13], [15], [16], including evidence of recombination among paralogs of *MATA-HMG* genes [47]. In the complete absence of sex, these genes would have been expected to become pseudogenes and unrecognizable as homologs [13], [14]. In *V. dahliae*, mating-type idiomorphs have been identified (see above) and evidence of repeat-induced point (RIP) mutations was found in a small subset of transposable elements in [48], [49]. RIP mutations prevent further transposition of repetitive elements in fungi but they only occur during meiosis [50], [51]. To our knowledge, no analyses have been published on meiosis-specific genes in *V. dahliae*.

The primary objective of this study was to test the hypothesis that clonal lineages of *V. dahliae* arose by recombination. In addition to lineages VCG2B^334^ and VCG2B^824^, and subgroups A and B of VCGs 2 and 4 discussed above, we looked for evidence of recombination within and between most of the known VCGs and lineages. A second objective was to determine the mating types of *V. dahliae* isolates in the major lineages. A third objective was to search for homologs of meiosis-specific genes in the *V. dahliae* genome. We used 26,748 SNPs obtained from 141 isolates of *V. dahliae* by genotyping by sequencing [52] to test for recombination. SNPs were distributed throughout the *V. dahliae* genome with known locations on chromosomes in a reference genome, allowing us to infer the number and locations of recombination events.

## Materials and Methods

### Isolates genotyped

We genotyped 141 *V. dahliae* isolates from diverse geographic and host origins, representative of all but one of the known *V. dahliae* VCGs (Table 1). These isolates were selected from the *V. dahliae* collections in the Department of Crop Protection, Institute for Sustainable Agriculture, CSIC, Córdoba, Spain and the Department of Plant Pathology and Environmental Microbiology, the Pennsylvania State University, USA. The selected isolates had previously been characterized for VCG and molecular markers, as well as virulence on their hosts, and have previously been used in published research (Table S1). Isolates were stored by covering cultures on plum-lactose-yeast extract agar (PLYA) [53], with sterile liquid paraffin [54] at 4°C in the dark.

**Table 1:** Sampling of *Verticillium dahliae* isolates genotyped from each vegetative compatibility group (VCG)^a.^

VCG	No. isolates	Countries of origin	Hosts of origin
1A	73	Spain, Greece, Israel, Turkey, USA, China	olive, cotton
1B	5	USA	Various woody plants
2A	13	Spain, Israel, USA	Artichoke, cotton, eggplant, olive, tomato
2B	29	Spain, Greece, Israel, Turkey,	Artichoke, cotton, olive, almond tree
4A	12	USA	potato, soil
4B	7	USA, Israel, Spain	cotton, olive, potato, soil
6	2	USA	pepper

a Complete list of isolates and their original sources can be found in Table S1.

No isolates of VCG3 and only two isolates of VCG6 were available to us at the time of this study; however, these VCGs are very rare [55]. We genotyped a large number of isolates of VCG1A because of their agricultural significance as highly virulent, defoliating pathogens of cotton and olive [23]. We used VCGs as a basis for choosing isolates to genotype because of their previous importance for describing population structure in *V. dahliae* before the availability of other markers, and because of their correlation with clonal lineages. In this study, we refer to lineages inferred from SNP genotyping as 2A, 4B, etc., after their respective VCGs, but we drop the VCG designation because lineages are defined by SNP haplotypes, not heterokaryon incompatibility.

### DNA preparation

Each isolate was cultured for DNA extraction as follows. Isolates were grown on modified sodium polypectate agar (SPA) selective for *Verticillium*; this medium contains streptomycin sulphate, chloramphenicol, and chorotetracycline HCl [56] to avoid bacterial contamination. Hyphae were transferred from these cultures to potato dextrose-agar (PDA) amended with 50 mg/L of chlorotetracycline, after which single conidia were transferred to new PDA plates. Cultures were grown in the dark at 25°C for 1 week to select isolates showing colonies typical of *V. dahliae*. Cultures were grown on PDA overlain with cellophane for 6–7 days before mycelium was harvested and lyophilized. Fifty mg lyophilized mycelia were ground to a fine powder in 2-ml tubes with glass beads in a Fast Prep Instrument (BIO 101, Carlsbad, CA). DNA was extracted from ground mycelia using the Kit i-Genomic Plant DNA Extraction Mini Kit (Intron Biotechnology, INC.). DNA purity and concentration were determined using a Nanodrop spectrophotometer ND-1000 (Nanodrop Technologies, Wilmington, DE) and by 1% agarose gel electrophoresis.

### Genotyping by sequencing

Purified DNA was submitted to the Cornell University Institute for Genomic Diversity (IGD) (http://www.igd.cornell.edu/index.cfm/page/GBS.htm) for genotyping by sequencing (GBS) as described by Elshire et al. [52]. In this method, a reduced-representation library is created by digesting genomic DNA with a restriction enzyme, ligating oligonucleotide adapters onto restriction fragments for PCR and sequencing, and sequencing the ends of the restriction fragments on an Illumina next-generation sequencing platform. Sequence tags were then aligned to the reference genome of isolate VdLs.17 [48], which is available online at http://www.broadinstitute.org. Only sequences that aligned to the reference genome could yield SNPs; all other sequences were discarded. SNPs were called based on differences among the isolates genotyped, not relative to the VdLs.17 genome sequence. Data were filtered by IGD for 80% coverage in all isolates per nucleotide site and a minimum minor allele frequency of 1%. Additional filtering was done using Tassel 4.0 (http://www.maizegenetics.net/), by recoding heterozygous sites (caused by sequencing or alignment errors) as missing data, and removing isolates with data at <90% of the scored nucleotide sites. Exploratory analysis was done by constructing a neighbor-joining tree [57] in MEGA 5.10 [58] to compare the genetic relationships among clonal lineages to those found in previous studies.

### Recombination analyses

We used Recombination Detection Program v.3.44 (RDP3) to identify unique recombination events [59]. RDP3 uses multiple analyses to detect recombination. All possible sets of three haplotypes (triplets) are examined to look for recombination. In closely related haplotypes, the same recombination events may be detected multiple times, but RDP3 scores only unique events. All recombination analyses were done using default settings in RDP3, including Bonferroni corrections for multiple tests. Recombination events were considered significantly more likely than mutation if they were detected by two or more different analyses in RDP3. Because we found large numbers of recombination events (see Results), we did not try to identify putative parents and recombinants.

Recombination analyses use only polymorphic nucleotide sites in sequence alignments, effectively ignoring all monomorphic sites. Therefore, we used SNP haplotype data with the rationale that SNPs ordered according to their positions on supercontigs in the VdLs.17 sequenced reference genome (hereafter referred to as contigs) are a proxy for actual sequence data. We used all the nucleotide sites called in the initial genotyping dataset for recombination analyses, which upon closer analysis included many monomorphic sites (see Results). We retained the full data set because of the constraint in RDP3 to have minimum sequence identity of 70%; the observed minimum identity among SNP haplotypes was approximately 80% when all sites were used.

For analysis of recombination among lineages, we used a subset of haplotypes to avoid duplication of nearly identical haplotypes. Analysis of highly similar haplotypes results in detecting the same recombination events multiple times, but does not increase the likelihood of detecting additional unique recombination events (RDP3 Instruction Manual). We used a minimum pairwise distance between haplotypes of 0.00017, (equal to a maximum similarity of 0.99983). This criterion was calculated from the formula in the RDP3 Instruction Manual: *Y* = 2 ln4*X*/*L*, where *Y* is the minimum pairwise distance between haplotypes, *X* is the number of haplotypes and *L* is the length of the sequence (in this case, the total number of SNPs). Recombination analyses were also done using a reduced set of isolates for each contig separately and for contigs on each of the eight chromosomes in the *V. dahliae* genome concatenated.

### Mating-type assays

The mating type idiomorph in each *V. dahliae* isolate was determined by multiplex PCR using primers pairs VdMAT1-1a/VdMAT1-1b and VdMAT1-2a/VdMAT1-2b [31], resulting in amplicons of approximately 400 bp or 600 bp in *MAT1-1* and *MAT1-2* idiomorphs, respectively. PCR reaction mixtures (20 µl) included 20 ng of fungal DNA template, 100 pmol of each primer and 10 µl of Premix Ex Taq Hot Start Version (Takara Bio). Amplifications were performed in a PTC-100 thermocycler (MJ Research, Inc., Watertown, MA) using the following conditions: denaturation at 94°C for 3 min followed by 30 cycles of 30 s at 94°C, 30 s at 65°C, 1 min at 72°C, and a final step of 72°C for 3 min. The PCR products were separated on 1% agarose gels stained with ethidium bromide and visualized under UV light. A set of reactions always included negative controls (no DNA), and positive controls using template DNA from *V. dahliae* isolates known to carry *MAT1-1* (isolate W83) or *MAT1-2* (isolate 461) idiomorphs [19].

### Homologs of meiosis-specific genes in *V. dahliae*

We searched for homologs of meiosis-specific genes in the *V. dahliae* genome of isolate VdLs.17 [48] using genes identified in *Aspergillus fumigatus* or *Saccharomyces cerevisiae* as homologs with known meiosis-specific function [14] (Table 2). Protein sequences for 11 meiosis-specific genes from the sequenced *A. fumigatus* genome of isolate Af293 were obtained from GenBank [60]. Because we could not find *HOP2* in the *A. fumigatus* genome, we used a sequence for this gene from *S. cerevisiae*. *V. dahliae* candidate genes were identified by blastp analysis through the Broad Institute website (http://www.broadinstitute.org). Candidate homologs were determined using reciprocal best hit analysis. Resulting proteins were then analyzed using the Conserved Domains tool on NCBI.

**Table 2:** Identification of homologs of meiosis-specific genes in the sequenced genome of *Verticillium dahliae* isolate VdLs.17.

Gene^a^	*Aspergillus fumigatus*Accession	*Verticillium dahlia*Accession	Total alignmentScore	Expect Value	% Identity	Protein Domains
*SPO11*	XP_747937.2	EGY19025	165	2e-50	32	Conserved
*REC8*	XP_747786	EGY21140	143	2e-39	27	Conserved
*RAD21*	XP_749714.1	EGY18368	447	e-152	47	Conserved
*RAD51*	XP_752407	EGY18462	653	0	89	Conserved
*MSH4*	XP_749950	EGY21332	627	3e-153	42	Partially Conserved
*MSH2*	XP_754634.1	EGY20237	1416	0	73	Conserved
*MSH5*	XP_752482.2	EGY18511	438	e-122	41	Partially Conserved
*MSH6*	XP_751995.1	EGY17510	1392	0	61	Conserved
*HOP1*	XP_751182.1	No homolog found	-	-	-	-
*HOP2*	EWH18245^b^	No homolog found	-	-	-	-
*MND1*	XP_751862	No homolog found	-	-	-	-
*DMC1*	XP_746712.1	No homolog found^c^	-	-	-	-

Protein sequences of 11 meiosis-specific genes in *Aspergillus fumigatus* [14] and one in *Saccharomyces cerevisiae* (*HOP2*) were used to search the *V. dahliae* genome using blastp. Accession numbers are from GenBank.

a Genes in *Saccharomyces cerevisiae* previously shown to function in meiosis [14].

b *HOP2* sequence from *S. cerevisiae* was used for searching the VdLs.17 genome.

c Although homologs are present, protein domain analyses suggests that the best hit is the related protein encoded by *RAD51*, not *DMC1*.

## Results

### SNP discovery

We detected a total of 74,146 putative SNPs from GBS that aligned with the reference genome of *V. dahliae*. After filtering out polymorphic sites caused by missing or ambiguous data, and isolates with too much missing data, we were left with 26,748 SNPs in 141 isolates (Table S2 and S3). The number of SNPs per contig is highly correlated with the length of the 52 contigs (*r* = 0.97, Table S4). Therefore, we conclude that SNPs identified by GBS are distributed throughout the *V. dahliae* genome, and therefore are appropriate for analyzing genome-wide patterns of recombination.

### Clonal population structure of *V. dahliae*

Neighbor-joining analysis on 26,748 SNPs resulted in a tree (Figure 1) with almost exactly the same topology as found by Collado-Romero et al. [18], who used maximum parsimony analysis on AFLP and multilocus sequence data. All clades and lineages mentioned hereafter had bootstrap support in the neighbor-joining analysis of greater than 99%. Neighbor-joining analysis shows a clear division of haplotypes into clades I and II defined by Collado-Romero et al. [18]. In the present analysis, we define subclade II-1 as comprising lineages 2A and 4B, and subclade II-2 as comprising lineages 2B^824^, 4A and 6. The close agreement between results from GBS and previous work confirms the markedly clonal structure of *V. dahliae* populations. The only discrepancy between assignment to VCG and SNP haplotypes were in three isolates previously determined to be in VCG1A (V1202, V1278 and V1487) that had SNP haplotypes in lineage 2A. VCG assays and GBS were repeated to confirm these discrepancies.

### Detection of recombination

Observation of haplotypes in intermediate positions between well-defined clades in the neighbor-joining tree suggests that they could be recombinants. Most prominently, we found an intermediate clade with haplotypes of two isolates in VCG2B between clades I and II; we named this lineage 2B^R1^ (Figure 1). Other putative recombinant haplotypes were less obvious in Figure 1; for example, lineage 6 appears to be a recombinant between lineages 2B^824^ and 4A. Haplotypes with similarities less than 0.99983 (the threshold calculated above in Methods section) compared to other haplotypes in the same lineage were also tested for recombination. In total, 26 haplotypes were used in recombination analyses among all lineages.

**Figure 2:**
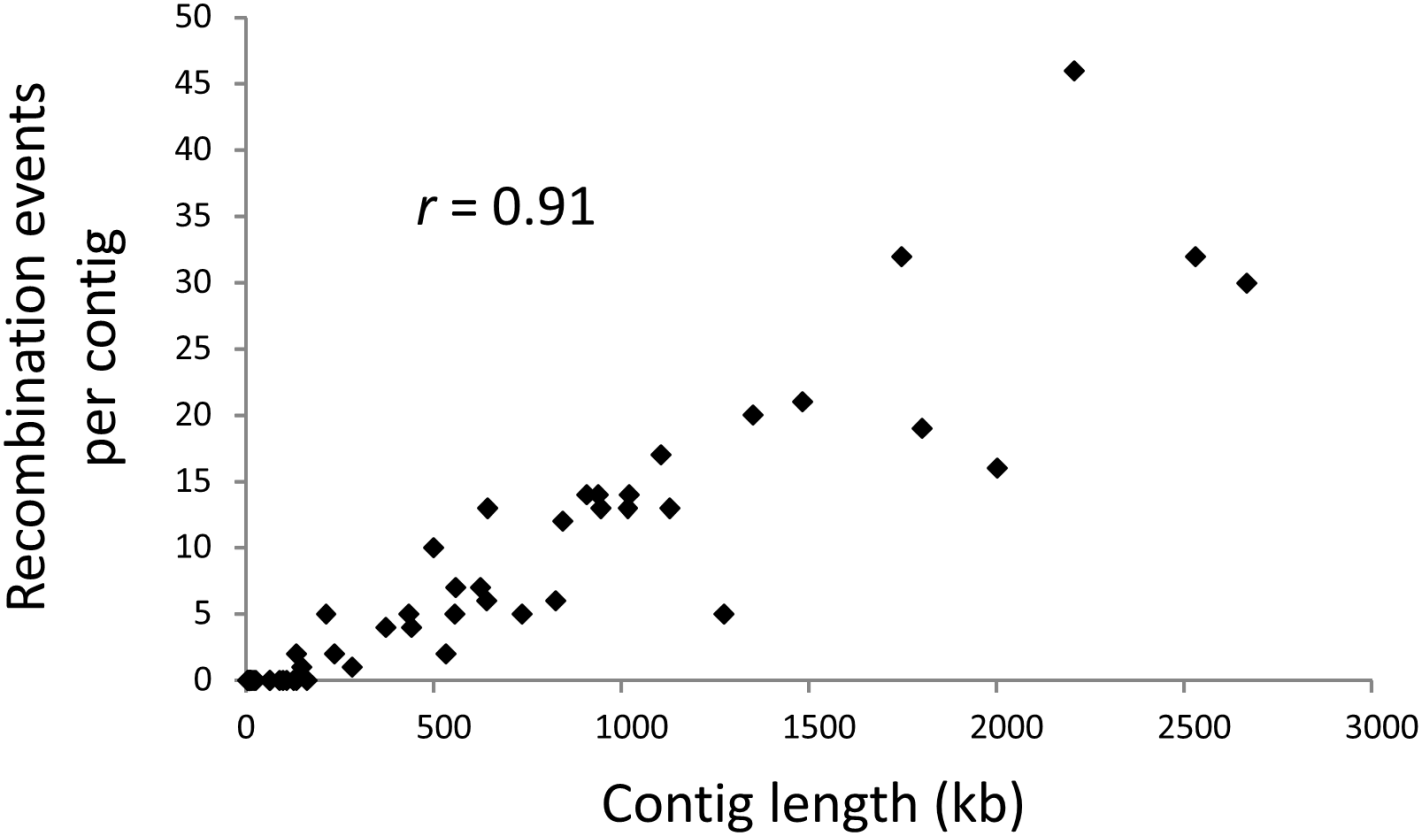
Correlation of number of recombination events detected per contig with contig length in *Verticillium dahliae* (*r* = 0.91).

We identified a total of 443 recombination events using RDP3 when all SNPs were concatenated into a single sequence (Table 3), and 411 for the sum of events within chromosomes when contigs were concatenated for each chromosome (Table S4). The total number of events within chromosomes increases to 432 when the 21 events detected within contigs not assigned to chromosomes were added. The two estimates are close enough that we are confident in using concatenated contigs for most subsequent analyses. We detected recombination within each of the eight chromosomes. The number of recombination events per chromosome ranged from 30 to 82 when haplotypes of all lineages were analyzed (Table S4). The number of recombination events per contig is highly correlated with the number of SNPs per contig (*r* = 0.95) and contig length (*r* = 0.91, Figure 2). A greater number of recombination events would be expected in longer contigs. Therefore, we conclude that recombination events are distributed throughout the genome.

**Table 3:** Recombination detected within and between clades of *Verticillium dahliae* shown in Figure 1 using the program RDP3 [59].

Clade	Lineages	Number of individualsin clade or lineage	Number of SNPsin lineage	Number of recombinationevents detected^a^
I, II and lineage 2B^R1^	All lineages	141 (26)^b^	26748 (26552)^c^	443
I & II	All lineages, excluding 2B^R1^	139 (25)	26406 (26210)	364
I	1A/1B & 2B^334^	92 (13)	4333 (4244)	22
	1A/1B & 2B^334^ (excludinghaplotype VEMS481)	91 (12)	4126 (4037)	0
	1A/1B	78	1040	2
	1A/1B (excluding haplotype VEMS481)	77	175	0
	2B^334^	14	62	0
II	II-1 & II-2	47 (14)	18374 (18165)	338
II-1	2A & 4B	20	5958	65
	2A & 4B (excluding haplotypes395, V496 and V498)	17	4492	0
	2A	13	3290	22
	2A (excluding haplotypes 395,V496 and V498)	10	92	0
	4B	7	94	0
II-2	2B^824^, 4A & 6	27	11000	90
	2B^824^ & 4A, excluding lineage 6	25	7348	4
	2B^824^ & 4A, excluding lineage 6& haplotype 320	24	7245	0
	2B^824^	13	212	0
	4A, including haplotype 320	12	144	0

SNP haplotypes were generated by genotyping by sequencing [52] by alignments of sequences to reference genome of isolate VdLs.17 [48].

a Number of recombination events was determined in RDP3 using default parameters for significant events.

b Number of haplotypes in subset used in RDP3 analysis is shown in parentheses; highly similar haplotypes were omitted because they add significantly to computation time but do not help to reveal unique recombination events.

c Number of SNPs in the full data set, with the number for subsets used in RDP3 in parentheses.

We tested for recombination within and between the clades and mapped the number of events hierarchically onto the neighbor-joining tree (Figure 1). As stated above, we identified a total of 443 recombination events among all clades combined (Table 3). Repeating the same analysis but excluding haplotypes in lineage 2B^R1^ resulted in 364 recombination events, 79 fewer than among all lineages. We interpret this to mean that 79 recombination events are attributable to the inclusion of lineage 2B^R1^ in the analysis. We used this same kind of reasoning to infer the number of recombination events associated with each lineage (Figure 1, Table 3).

**Figure 3:**
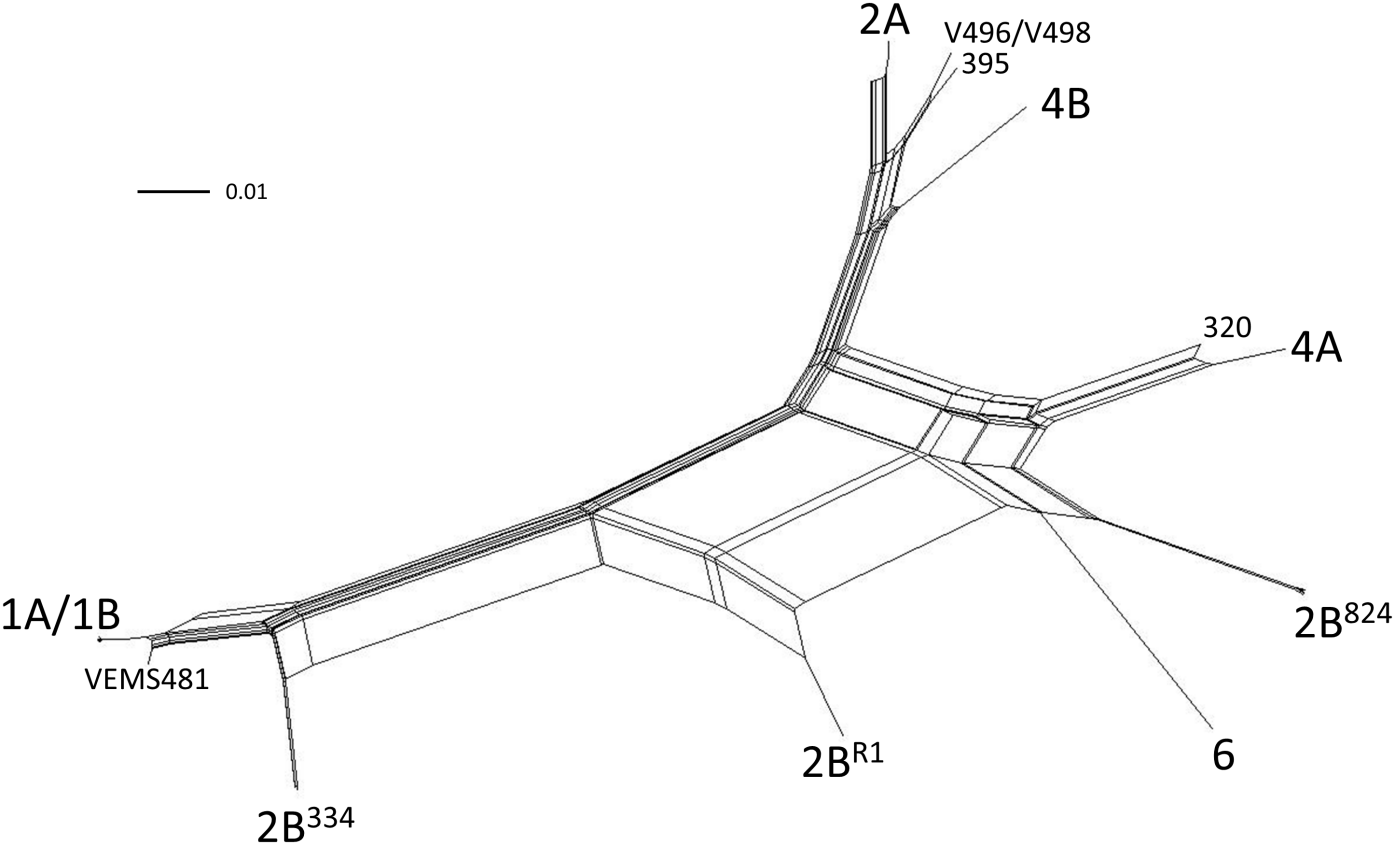
A neighbor-net network of all lineages and recombinant haplotypes of *Verticillium dahliae*, based on 26,748 SNPs. The prevalence of closed loops in the network is consistent with other analyses showing extensive recombination among clonal lineages (Figure 1; Table 3). The positions are shown for all lineages, plus individual haplotypes identified as recombinants (isolates VEM481, V496/V498, 395 and 320).

From these analyses we conclude that recombination occurred between clades. Because of the phylogenetic incompatibility inherent in recombination, a bifurcating tree, such as the one generated from neighbor-joining analysis (Figure 1), is not an appropriate reconstruction of phylogeny [61]. As an alternative, we show the genetic relationships among lineages and other recombinant haplotypes in a neighbor-net network constructed using SplitsTree version 4.13.1 [62]. Closed loops in haplotype networks (Figure 3) are indicative of the uncertainty of phylogenetic reconstructions caused by recombination among lineages [61]. Moreover, the PHI test, based on phylogenetic incompatibility [63], implemented in SplitsTree detected significant recombination overall (*P*<0.001).

### Nucleotide variation in nonrecombining lineages

The number of segregating sites (*S*) and nucleotide diversity (π) within each lineage were determined using Tassel 4.0 for each of the nonrecombining clades (recombinant haplotypes were excluded from lineages 1A/1B, 2A and 4A) (Table 4). Estimates of π ranged from 0.00022 to 0.00026 for lineages 1A/1B, 2A and 2B^334^. Lineages 2B^824^ and 4B were more diverse, with estimates of π of 0.00121 and 0.00064, respectively, which are approximately two to five times greater than the diversity found in other lineages. Sampling from broad geographic locations and multiple hosts may have contributed to high diversity in these latter two lineages, although comparable broad sampling was done for lineages 1A/1B and 2A, both of which have lower diversity (Table 4). The lowest diversities were found in lineages 2B^R1^, 4A and 6; however, we genotyped only two isolates in lineages 2B^R1^ and 6, and lineage 4A is found almost exclusively on potato in North America [26].

**Table 4:** Nucleotide diversity within nonrecombining lineages of *Verticillium dahliae* based on 26,748 SNPs from genotyping by sequencing [52].

Clade	Lineages	Number ofindividuals, *N*	Number ofsegregating sites, *S*	Nucleotide diversity, π
I	1A/1B (excluding isolate VEMS481)	77	175	0.00023
	2B^334^	14	61	0.00022
2B^R1^	2B^R1^	2	1	<0.00001
II-1	2A (excluding isolates 395,V496 and V498)	10	92	0.00026
	4B	7	94	0.00064
II-2	2B^824^	13	212	0.00121
	4A	12	144	0.00036
	6	2	9	0.00013

Isolates with recombinant haplotypes in lineages 1A/2B and 2A (Figure 1) were excluded because they would increase the diversity within each lineage, but not by mutation.

### Distribution of mating types

All but three isolates we genotyped have mating type *MAT1-2*. Both isolates in lineage 6 and isolate 395 in lineage 2A have mating-type *MAT1-1*. Interestingly, these *MAT1-1* isolates were shown to be the result of recombination (Table 3).

### Homologs of meiosis-specific genes

We found eight homologs of the 11 meiosis-specific genes in *A. fumigatus* and one in *S. cerevisiae* (Table 2). For all eight, protein domains were at least partially conserved with *A. fumigatus* and *S. cerevisiae*.

## Discussion

We present evidence that recombination occurred extensively throughout the genome of *V. dahliae*. Many unique recombination events could be detected between clades and between lineages within clades, with little evidence of recombination within lineages. Several recombinant haplotypes were identified that are likely to have arisen by recombination between closely related lineages, or their progenitors. The majority of the recombination events, however, could not be ascribed to specific recombinant haplotypes or lineages; nor could we identify putative parental haplotypes with any confidence. The lack of resolution in identifying putative parent haplotypes is most likely caused by multiple recombination events among clades, obscuring the specific details of individual events [64]. Furthermore, it is highly likely we did not sample parental haplotypes, but instead genotyped their descendants, some of which may have recombined multiple times in their evolutionary history, making the reconstruction of specific recombination events difficult, if not impossible. Nonetheless, we inferred recombinant lineages in three instances based on the history and distribution of extant lineages derived from extensive surveys of *V. dahliae* populations [17], [18], [19], [26], [39], [40], [41], [55], [65], [66], [67], [68], [69], [70]. First, lineage 6 is interpreted as a recombinant between lineages 2B^824^ and 4A (Figure 1, Table 3), both of which have been found commonly in North America [65], [71]. Lineage 6 has only been found recently in a few isolates from peppers in California [67]. Second, we interpret lineage 2B^R1^ as arising by recombination between clades I and II, both of which are widespread geographically. Lineage 2B^R1^ comprises two isolates from olive in Spain, but this lineage had never been described before this study. Finally, we speculate that lineage 2B^334^ arose by recombination between lineage 1A/1B and a lineage in clade II, most likely lineage 2B^824^, which is in the same VCG. Isolates in lineage 2B^334^ have only been found recently infecting artichoke in east-central Spain; isolates in lineages 1A/1B and VCG2B^824^ were also found infecting artichoke in some fields [40], [66]. We do not know how long these recombinant lineages were present in *V. dahliae* populations before being sampled. Overall, however, clonal lineages of *V. dahliae* appear to have arisen by recombination and contributed to the emergence of new genotypes, sometimes with adaptation to new hosts.

Despite the evidence of recombination and the inference that lineages of *V. dahliae* originally arose by recombination, extant populations are markedly clonal, as shown by four lines of evidence. First, individuals with nearly identical SNP haplotypes were sampled repeatedly, often from different hosts, and/or from different continents. For example, isolates in clonal lineage 1A/1B from Europe, North America and China have nearly identical haplotypes. Second, clonal lineages defined by 26,748 SNPs in this study correlate closely to those defined previously by VCGs, AFLPs and sequences of various genes [18], [41]. Third, all but three of the isolates we sampled have the same mating type (*MAT1-2*), as found previously, where *MAT1-1* is rare [19], [30],[31],[32]. In ascomycete fungi, both mating-type idiomorphs are required for successful mating, either in the same genome in homothallic species, or in different individuals, each with one of the two mating types, in heterothallic species [33], [34]. Fourth, recent evidence of extensive chromosome rearrangements in *V. dahliae* suggests that meiosis may be impaired during sexual reproduction [35], [36], as discussed further below. The presence of a finite and stable number of clonal lineages makes it unlikely that *V. dahliae* populations experience frequent recombination between lineages at the present time. Nonetheless, new VCGs/lineages are occasionally found, e.g., lineage 6/VCG6, as described above. Overall, we conclude that recombination may not occur frequently in *V. dahliae*, but it has the potential to give rise to new clonal lineages.

A crucial question that remains to be answered is the mechanism of recombination in *V. dahliae*. Although there is no known sexual stage for *V. dahliae*, the presence of both mating-type idiomorphs and homologs of meiosis-specific genes strongly suggest that *V. dahliae* has either lost sexuality recently or maintained the potential to reproduce sexually. This type of evidence has been used previously to infer the potential for sex in other fungi, most notably in the Glomeromycota [13], [14], [16]. Most individuals of *V. dahliae* in this study have mating type *MAT-2*. The three individuals with *MAT1-1* were identified independently as recombinants. Mating type segregates in 1∶1 ratios during sexual reproduction in heterothallic ascomycetes. By chance, three recombinants have the less common mating type, *MAT1-1*, whereas other recombinants haplotypes have *MAT1-2*.

We also found homologs of eight meiosis-specific genes in the reference genome of *V. dahliae* isolate VdLs.17. All of these homologs had conserved or partially conserved protein structures as those found in *A. fumigatus* and *S. cerevisiae* (Table 2). Such conservation of structure might indicate that these genes have retained the potential to function in sexual reproduction. If sexuality had been lost a long time ago, we would expect these genes to accumulate mutations in coding regions as if they were pseudogenes. These results, however, must be interpreted cautiously. The asexual, human pathogenic fungus *Candida albicans* has been shown to complete the sexual cycle, albeit abnormally [72]. However, the genes involved in sexuality in *C. albicans* may be conserved because they have been co-opted for other processes, such as pathogenicity [73]. Therefore, the presence of functional homologs does not necessarily indicate that *V. dahliae* can reproduce sexually. Furthermore, four meiosis-specific genes (*HOP1*, *HOP2*, *MND1*, and *DMC1*) were not found in VdLs.17. However, these four genes are not found in the genomes of *N. crassa*, *Sordaria macrospora* or *Podospora anserina* in the Sordariomycetes (like *V. dahliae*), or in *Ustilago maydis* (Basidiomycota) [16], yet all of these species reproduce sexually. Much of the work determining which genes are required for meiosis has been done in model species, particularly yeasts [74]. Thus, the absence of genes shown to be required for meiosis in other species does not necessarily indicate an inability to reproduce sexually. Despite the uncertainties associated with these genes, their presence in genomes of asexual species has been widely interpreted as evidence of the potential for meiosis and sexual reproduction [13], [14], [16].

Some authors have speculated that meiosis is unlikely to occur in *V. dahliae* because of extensive genomic rearrangements [35], [36]. Chromosome inversions suppress crossing over only within the inverted region–such recombinants are lethal–but otherwise meiosis can occur normally. Translocations, by contrast, result in abnormal chromosome alignment during synapsis and a high frequency of inviable gametes. Despite reduced fertility, viable recombinant gametes may be produced, albeit at a lower frequency. In fact, many sexually reproducing fungi exhibit considerable chromosome length polymorphisms caused by translocations [37], [38]. Multiple translocations, as observed in *V. dahliae* [35], would reduce the probability of viable recombinant offspring, but they may not completely exclude the possibility of rare sexual reproduction and recombination by meiosis. If chromosome rearrangements prevent successful sexual reproduction between lineages [35], [36], then the recombination we detected either occurred before the rearrangements, or it occurred by non-meiotic mechanisms. However, a mechanism like horizontal gene transfer would be unlikely to occur extensively enough to account for the evidence of recombination occurring throughout the genome (Figure 2). Meiosis prior to rearrangements seems like a more parsimonious explanation.

**Figure 4:**
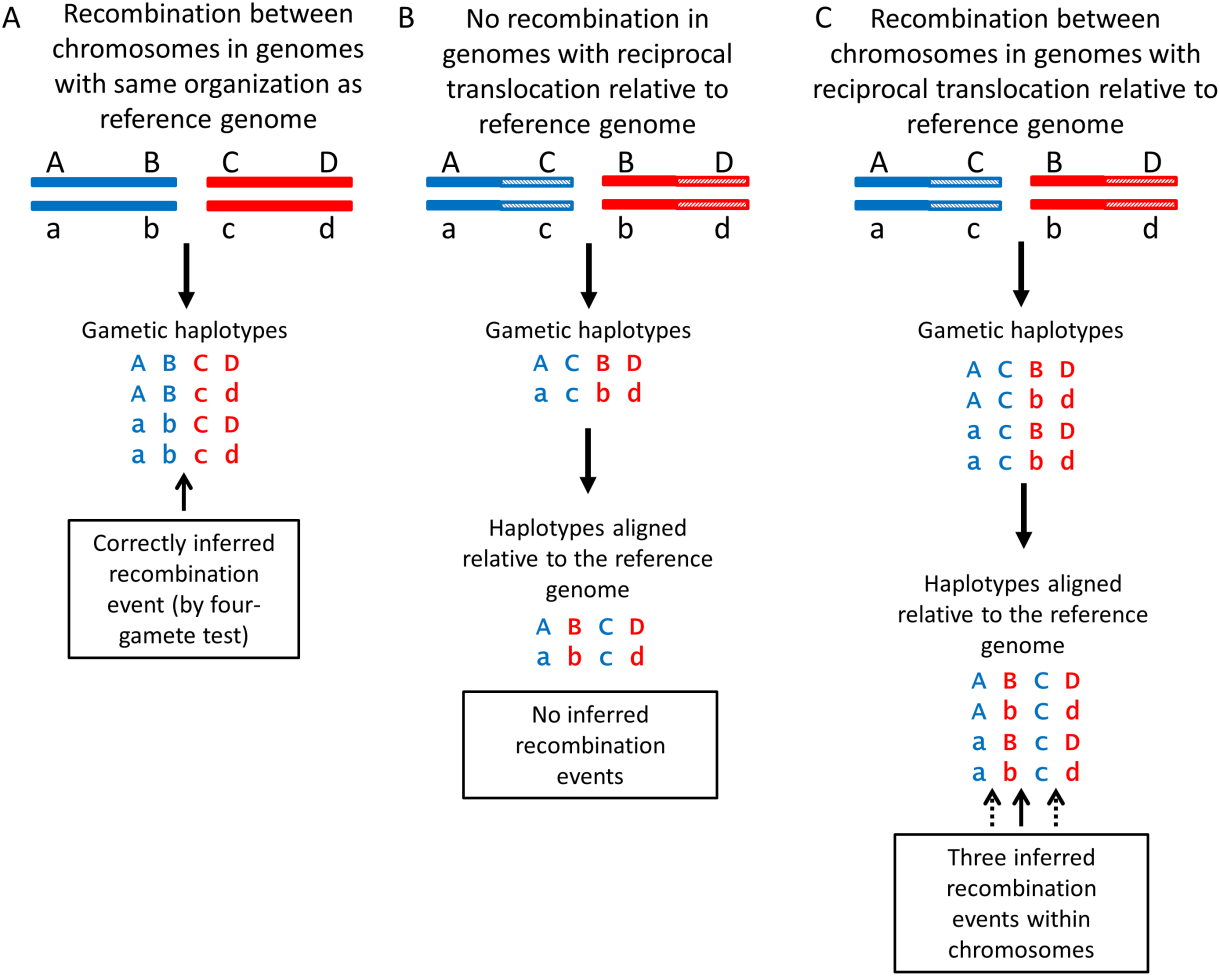
Confounding effects of chromosome rearrangements on the estimation of the number and location of recombination events when aligning SNPs to a single, haploid reference genome. In this example, recombination occurs between haplotypes *ABCD* and *abcd*. **A.** Pairings of two homologous chromosomes (blue and red) during meiosis in haploid genomes with the same organization as the reference genome. The four haplotypes of the gametes are shown after recombination between chromosomes by assortment (assuming no crossing over within chromosomes). Recombination is correctly inferred as occurring between *AB* and *CD* based on the presence of four gametic haplotypes. **B.** Mating between individuals with the same reciprocal translocation relative to the reference genome, but without recombination within or between the two chromosomes. No recombination is inferred: only two gametic haplotypes are present. **C.** Recombination between chromosomes in individuals with the same reciprocal translocation relative to the reference genome. When the gametic haplotypes are aligned to the reference genome, the gene order changes and three recombination events are inferred, two of which are incorrectly inferred as occurring within chromosomes of the reference genome (dashed arrows).

Parasexuality is an alternative, non-meiotic mechanism of recombination that has been demonstrated in laboratory studies of many ascomycetes [75], [76], including *V. dahliae* [77]. However, parasexuality has never convincingly been demonstrated to occur in nature (but see [78]). A crucial requirement of parasexuality is that individuals form stable heterokaryons to initiate the process [75], [76]. Individuals in different VCGs cannot form stable heterokaryons [24], [25] and therefore are highly unlikely to recombine parasexually. Heterokaryosis can result from mutation within an individual, but the lack of polymorphism observed within lineages would argue against this hypothesis for explaining the recombination detected. Recombination between lineages represented by different VCGs, therefore, are not likely to be the result of parasexuality. Recombination can potentially occur by parasexuality within VCGs, but we found no evidence to support this hypothesis. For example, 20 of the 24 recombination events detected among the three lineages of VCG2B (2B^334^, 2B^824^ and 2B^R1^) occurred within chromosomes, with at least one event on each of the eight chromosomes. In lineage 2A, 22 recombination events were found within seven of the eight chromosomes. All recombination within lineage 2A was attributable to three recombinant haplotypes (isolates 395, V496 and V498), all of which were also associated with recombination between lineages 2A and 4B, mostly likely by sexual reproduction between VCGs. Within-chromosome recombination by mitotic crossing over is rare in parasexuality [76], making these results more consistent with the hypothesis that recombination occurred by meiosis than by parasexuality. Alternatively, these arguments based on detection of within-chromosome recombination may be confounded by chromosome rearrangements, which are common in *V. dahliae* [35]. When recombination occurs in a genome that has a different organization from the reference genome because of rearrangements, alignment of SNPs to the reference genome may inflate the number of within-chromosome recombination events detected (Figure 4). We fully acknowledge this source of bias in our estimation of recombination events. However, it is crucial to emphasize that without recombination, either within or between chromosomes, chromosome rearrangements per se would not affect the detection of recombination in our analyses. Rearrangements only affect the estimation of the number of events and whether recombination is inferred to have occurred by crossing over or by assortment of chromosomes. Therefore we cannot definitively rule out parasexuality from the large number of recombination events within chromosomes, but neither do we find evidence in support of parasexuality.

We cannot determine the time since the origin of clonal lineages with any confidence using methods of molecular evolution. Many SNPs are shared between lineages because of recombination, whereas dating techniques are typically based on fixed differences between taxa. Comparisons of nucleotide diversity in nonrecombining lineages (i.e., with recombinant haplotypes excluded) could potentially show their relative ages. However, estimates of nucleotide diversity are confounded by differences in sampling among lineages. Sample sizes and their geographic and host distributions varied among lineages, which are likely to affect estimates of diversity. Dating techniques depend on estimates of mutation rates–which are not known for most fungi. More importantly, molecular clocks are based on assumptions of neutrality. The small number of clonal lineages with widespread geographic distributions, together with limited diversity within lineages, is consistent with a history of selective sweeps. Because *V. dahliae* is primarily clonal, the entire genome is effectively linked and affected by hitchhiking selection. Therefore, reduced diversity within lineages could be caused either by hitchhiking selection or by a recent origin after which few neutral mutations have occurred.

The population structure of *V. dahliae* determined by 26,748 SNPs is highly clonal, with the exception of a small number of recombinant haplotypes (Figure 1). This result confirms those of previous studies done with AFLPs or multilocus sequencing [18], [41]. Because we used such a large number of SNPs, evenly distributed throughout the genome, our analysis provides a definitive assessment of population structure. The high-resolution genotyping afforded by GBS also allowed us to address questions of recombination on a fine scale. Furthermore, we now have a large number of candidate SNPs unique to each lineage that can be used to develop diagnostic markers. For instance, individuals in VCG1A are highly virulent, cause rapid defoliation of cotton and olive, and are of great agricultural interest [23]. Previous attempts to develop markers specific to VCG1A, however, have not been specific enough [40], [68], [79]. SNPs unique to VCG1A identified in this study (data not shown) are now available for developing diagnostic tools. Overall, GBS is a powerful genotyping method in this system and should prove to be a valuable method for other clonal fungi.

The samples of *V. dahliae* used in this study reflect the agricultural bias of the plant pathologists interested in this fungus. Sampling has been done almost exclusively from the roots or shoots of infected crop plants, although we included a small number of isolates from agricultural soil. However, an important question about the biology of *V. dahliae* is whether its clonal population structure is a consequence of agriculture. *V. dahliae* has a broad host range, with some lineages better adapted to some host plants than others [19], [40], [66], [69], [70]. For example, individuals of VCG4A are almost always associated with potatoes, and only found—so far—in North America [71]. Agriculture may have at least two profound effects on the population structure of *V. dahliae*. First, the presence of large, stable, genetically uniform host populations may select for genotypes that are adapted to agricultural crops. We speculate that at some time in the past sexual reproduction and recombination resulted in a small number of genotypes with high fitness on particular cultivated host species or cropping systems, which then increased to high frequencies asexually in selective sweeps. This phenomenon has been referred to as an “epidemic” population structure in bacterial populations [80]. Among plant-pathogenic fungi, epidemic population structures have been described for fungi such as *Sclerotinia sclerotiorum* or *Sclerotium cepivorum* which have clones that originally arose by recombination [81], [82]. In *V. dahliae*, new lineages are not found frequently, but they appear to have arisen by recombination, perhaps in recent times. The second major effect of agriculture on the population structure of *V. dahliae* is that the most common haplotypes have been widely dispersed geographically, most likely on seed or asexual planting material, or in the international trade in agricultural commodities [83], [84], [85], [86]. This has resulted in worldwide distributions of some clonal lineages.

## Conclusions

Populations of *V. dahliae* sampled from agricultural crops are highly clonal in structure, but extant lineages appear have arisen by recombination from sexual ancestors. In addition, the presence of both mating-type idiomorphs and conservation of meiosis-specific genes suggests that *V. dahliae* may have retained the ability to reproduce sexually. The relatively recent emergence of some clonal lineages found in limited geographic locations, only on single host species, suggests that recombination may still occur. Alternatively, as with other fungi, *V. dahliae* may have lost sexuality only recently. The loss of sexuality may have been favored by modern agricultural conditions where well-adapted genotypes encounter large, stable, genetically uniform host populations. To fully understand the population biology and sexuality in *V. dahliae*, studies needs to be done in populations associated with nonagricultural hosts or in asymptomatic agricultural hosts where it is an endophyte [87].

## Supporting Information

Table S1 ***Verticillium dahliae*** **isolates genotyped.** Complete list of isolates genotyped, with country and host of origin, original published names and sources, and lineage and mating-type data.
(DOCX)

Table S2 **SNP data in hapmap format.** Data for 26,748 SNPs are provided in hapmap format which can be read into Tassel 4.0 (http://www.maizegenetics.net/) to find the locations relative to the reference genome of isolate VdLs.17.
(TXT)

Table S3 **SNP data in Phylip format.** Data for 26,748 SNPs are provided in Phylip interleaved format for analyses standard software.
(PHY)

Table S4 **Number of SNPs and recombination events per contig.** Number of SNPs and recombination events for each supercontig relative to the *Verticillium dahliae* reference genome for isolate VdLs.17.
(DOCX)
