# Supplementary material for: Recombination between Clonal Lineages of the Asexual Fungus Verticillium dahliae Detected by Genotyping by Sequencing
Source: PLoS One. 2014 Sep 2;9(9):e106740. doi: 10.1371/journal.pone.0106740 (PMC4152335; doi:10.1371/journal.pone.0106740)
Supplement: Table S1 — Verticillium dahliae isolates genotyped. Complete list of isolates genotyped, with country and host of origin, original published names and sources, and lineage and mating-type data. (DOCX) [file pone.0106740.s001.docx]

Table S1. Isolates of *Verticillium dahliae* genotyped.

| VCG/ lineage | Mating type | Isolate | Host | Country | Original isolate name | References |
| --- | --- | --- | --- | --- | --- | --- |
| 1A | 1-2 | 1NK2_1 | Cotton | China | 1NK2_1 | Xu et al. 2012 [[1](#_ENREF_1)] |
| 1A | 1-2 | 4TM6_15 | Cotton | China | 4TM6_15 | Xu et al. 2012 [[1](#_ENREF_1)] |
| 1A | 1-2 | DX-2 | Cotton | USA | DX_2 | Joaquim, 1989 [[2](#_ENREF_2)] |
| 1A | 1-2 | T9 | Cotton | USA | T9 | Joaquim & Rowe [[3](#_ENREF_3)] |
| 1A | 1-2 | V1124 | Olive | Spain | VOR196I | Jiménez-Díaz et al. [[4](#_ENREF_4)] |
| 1A | 1-2 | V1143 | Olive | Spain | VOR231I | Jiménez-Díaz et al. [[4](#_ENREF_4)] |
| 1A | 1-2 | V117 | Cotton | Spain | V117I | Pérez-Artés et al. [[5](#_ENREF_5)], Korolev et al. [[6](#_ENREF_6)], Korolev et al. [[7](#_ENREF_7)], Collado-Romero et al. [[8](#_ENREF_8)] |
| 1A/2A^a^ | 1-2 | V1202 | Olive | Spain | VOR310I | Jiménez-Díaz et al. [[4](#_ENREF_4)] |
| 1A | 1-2 | V1234 | Olive | Spain | VOR346I | Jiménez-Díaz et al. [[4](#_ENREF_4)] |
| 1A/2A | 1-2 | V1278 | Olive | Spain | VOR390I | Jiménez-Díaz et al. [[4](#_ENREF_4)] |
| 1A | 1-2 | V1294 | Olive | Spain | VOR406I | Jiménez-Díaz et al. [[4](#_ENREF_4)] |
| 1A | 1-2 | V1329 | Olive | Spain | VOR910I | Jiménez-Díaz et al. [[4](#_ENREF_4)] |
| 1A | 1-2 | V1337 | Olive | Spain | VOR918I | Jiménez-Díaz et al. [[4](#_ENREF_4)] |
| 1A | 1-2 | V1340 | Olive | Spain | VOR453I | Jiménez-Díaz et al. [[4](#_ENREF_4)] |
| 1A | 1-2 | V1344 | Olive | Spain | VOR921I | Jiménez-Díaz et al. [[4](#_ENREF_4)] |
| 1A | 1-2 | V135 | Olive | Spain | V135I | Pérez-Artés et al. [[5](#_ENREF_5)], Collado-Romero et al. [[8](#_ENREF_8)] |
| 1A | 1-2 | V136 | Olive | Spain | V136I | Pérez-Artés et al. [[5](#_ENREF_5)], Collado-Romero et al. [[8](#_ENREF_8)] |
| 1A | 1-2 | V1365 | Olive | Spain | VOR783I | Jiménez-Díaz et al. [[4](#_ENREF_4)] |
| 1A | 1-2 | V138 | Cotton | Spain | V138I | Pérez-Artés et al. [[5](#_ENREF_5)], Korolev et al. [[6](#_ENREF_6)], Korolev et al. [[7](#_ENREF_7)], Collado-Romero et al. [[8](#_ENREF_8)], Collado-Romero et al. [[9](#_ENREF_9)] |
| 1A | 1-2 | V1477 | Olive | Spain | VOR589I | Jiménez-Díaz et al. [[4](#_ENREF_4)] |
| 1A/2A | 1-2 | V1481 | Olive | Spain | VOR593I | Jiménez-Díaz et al. [[4](#_ENREF_4)] |
| 1A | 1-2 | V151 | Olive | Spain | V151I | Pérez-Artés et al. [[5](#_ENREF_5)], Collado-Romero et al. [[8](#_ENREF_8)] |
| 1A | 1-2 | V1528 | Olive | Spain | VOR604I | Jiménez-Díaz et al. [[4](#_ENREF_4)] |
| 1A | 1-2 | V153 | Olive | Spain | V153I | Pérez-Artés et al. [[5](#_ENREF_5)], Collado-Romero et al. [[8](#_ENREF_8)] |
| 1A | 1-2 | V1667 | Olive | Spain | VOR743I | Jiménez-Díaz et al. [[4](#_ENREF_4)] |
| 1A | 1-2 | V1705 | Olive | Spain | VOR793I | Jiménez-Díaz et al. [[4](#_ENREF_4)] |
| 1A | 1-2 | V175 | Cotton | Spain | V175I | Collado-Romero et al. [[8](#_ENREF_8)] |
| 1A | 1-2 | V177 | Cotton | Spain | V177I | Pérez-Artés et al. [[5](#_ENREF_5)], Korolev et al. [[6](#_ENREF_6)], Collado-Romero et al. [[8](#_ENREF_8)] |
| 1A | 1-2 | V180 | Cotton | Spain | V180I | Pérez-Artés et al. [[5](#_ENREF_5)], Korolev et al. [[6](#_ENREF_6)], Collado-Romero et al. [[8](#_ENREF_8)] |
| 1A | 1-2 | V181 | Cotton | Spain | (X) V181I | Korolev et al. [[6](#_ENREF_6)], Collado-Romero et al. [[8](#_ENREF_8)], Collado-Romero et al. [[9](#_ENREF_9)] |
| 1A | 1-2 | V184 | Cotton | Spain | V184I | Pérez-Artés et al. [[5](#_ENREF_5)], Korolev et al. 2001, |
| 1A | 1-2 | V224 | Cotton | Spain | V224I | Korolev et al. [[6](#_ENREF_6)] |
| 1A | 1-2 | V231 | Cotton | Spain | V231I | Korolev et al. [[6](#_ENREF_6)] |
| 1A | 1-2 | V314 | Olive | Spain | (X) V314I | Collado-Romero et al. [[8](#_ENREF_8)] |
| 1A | 1-2 | V44 | Cotton | USA | V_44 | Joaquim & Rowe [[3](#_ENREF_3)] |
| 1A | 1-2 | V458 | Cotton | Israel | cot228 | Korolev et al. [[7](#_ENREF_7)] |
| 1A | 1-2 | V461 | Cotton | Israel | cot239 | Korolev et al. [[7](#_ENREF_7)] |
| 1A | 1-2 | V464 | Cotton | Israel | cot308 | Korolev et al. [[7](#_ENREF_7)] |
| 1A | 1-2 | V465 | Cotton | Israel | cot350 | Korolev et al. [[7](#_ENREF_7)] |
| 1A | 1-2 | V466 | Cotton | Israel | cot351 | Korolev et al. [[7](#_ENREF_7)] |
| 1A | 1-2 | V467 | Cotton | Israel | cot352 | Korolev et al. [[7](#_ENREF_7)] |
| 1A | 1-2 | V468 | Cotton | Israel | cot354 | Korolev et al. [[7](#_ENREF_7)] |
| 1A | 1-2 | V469 | Cotton | Israel | cot356 | Korolev et al. [[7](#_ENREF_7)] |
| 1A | 1-2 | V470 | Cotton | Israel | cot357 | Korolev et al. [[7](#_ENREF_7)] |
| 1A | 1-2 | V610 | Cotton | Spain | V610I | Collado-Romero et al. [[8](#_ENREF_8)] |
| 1A | 1-2 | V640 | Cotton | Greece | (X) V640I | Collado-Romero et al. [[8](#_ENREF_8)], Collado-Romero et al. [[9](#_ENREF_9)] |
| 1A | 1-2 | V641 | Cotton | Greece | (X)V641I | Collado-Romero et al. [[8](#_ENREF_8)], Collado-Romero et al. [[9](#_ENREF_9)] |
| 1A | 1-2 | V642 | Cotton | Greece | V642I | Collado-Romero et al. [[8](#_ENREF_8)] |
| 1A | 1-2 | V643 | Cotton | Greece | V643I | Collado-Romero et al. [[8](#_ENREF_8)] |
| 1A | 1-2 | V646 | Cotton | Greece | V646I | Collado-Romero et al. [[8](#_ENREF_8)] |
| 1A | 1-2 | V662 | Cotton | Greece | V662I | Collado-Romero et al. [[8](#_ENREF_8)] |
| 1A | 1-2 | V663 | Cotton | Greece | (X)V663I | Collado-Romero et al. [[8](#_ENREF_8)], Collado-Romero et al. [[9](#_ENREF_9)] |
| 1A | 1-2 | V667 | Cotton | Greece | V667I | Collado-Romero et al. [[8](#_ENREF_8)] |
| 1A | 1-2 | V809 | Cotton | Greece | (X)V809I | Collado-Romero et al. [[9](#_ENREF_9)] |
| 1A | 1-2 | V816 | Cotton | Spain | V816I | Collado-Romero et al. [[9](#_ENREF_9)] |
| 1A | 1-2 | VEMS481 | Cotton | USA | V_EMS(481) | Joaquim, 1989 [[2](#_ENREF_2)] |
| 1A* | 1-2 | V1859 | Olive | Turkey | OVd074 | Dervis et al. [[10](#_ENREF_10),[11](#_ENREF_11)] |
| 1A* | 1-2 | V1860 | Olive | Turkey | OVd75 | Dervis et al. [[10](#_ENREF_10)] |
| 1A* | 1-2 | V1861 | Olive | Turkey | OVd076 | Dervis et al. [[10](#_ENREF_10),[11](#_ENREF_11)] |
| 1A* | 1-2 | V1865 | Olive | Turkey | OVd079 | Dervis et al. [[10](#_ENREF_10),[11](#_ENREF_11)] |
| 1A* | 1-2 | V1870 | Olive | Turkey | OVd119 | Dervis et al. [[10](#_ENREF_10)], Dervis et al. [[11](#_ENREF_11)] |
| 1A* | 1-2 | V1871 | Olive | Turkey | OVd135 | Dervis et al. [[10](#_ENREF_10)] |
| 1A* | 1-2 | V1880 | Olive | Turkey | OVd166 | Dervis et al. [[10](#_ENREF_10),[11](#_ENREF_11)] |
| 1A* | 1-2 | V1891 | Olive | Turkey | OVd194 | Dervis et al. [[10](#_ENREF_10)] |
| 1A* | 1-2 | V1900 | Olive | Turkey | OVd249 | Dervis et al. [[10](#_ENREF_10),[11](#_ENREF_11)] |
| 1A* | 1-2 | V1904 | Olive | Turkey | OVd248 |  |
| 1A* | 1-2 | V1905 | Olive | Turkey | OVd251 | Dervis et al. [[10](#_ENREF_10)] |
| 1A* | 1-2 | V1906 | Olive | Turkey | OVd253 | Dervis et al. [[10](#_ENREF_10)] |
| 1A* | 1-2 | V1908 | Olive | Turkey | OVd263 |  |
| 1A* | 1-2 | V1969 | Olive | Turkey | OVd81 | Dervis et al. [[10](#_ENREF_10)] |
| 1A* | 1-2 | V278 | Cotton | Israel | cot200 | Korolev et al. [[7](#_ENREF_7)] |
| 1A* | 1-2 | V454 | Cotton | Israel | cot213 | Korolev et al. [[7](#_ENREF_7)] |
| 1A* | 1-2 | V462 | Cotton | Israel | cot242 | Korolev et al. [[7](#_ENREF_7)] |
| 1A* | 1-2 | V666 | Cotton | Greece | (X) V666I | Collado-Romero et al. [[8](#_ENREF_8)], Collado-Romero et al. [[9](#_ENREF_9)] |
| 1A* | 1-2 | V669 | Olive | Turkey | (X) V669I | Collado-Romero et al. [[8](#_ENREF_8)], Collado-Romero et al. [[9](#_ENREF_9)] |
| 1B | 1-2 | V517 | Woody plants | USA | (X) V517II and I | Collado-Romero et al. [[8](#_ENREF_8)], Collado-Romero et al. [[9](#_ENREF_9)] |
| 1B | 1-2 | V518 | Woody plants | USA | (X) V518II and I | Collado-Romero et al. [[8](#_ENREF_8)], Collado-Romero et al. [[9](#_ENREF_9)] |
| 1B | 1-2 | V606 | Woody plants | USA | V606I | Collado-Romero et al. [[8](#_ENREF_8)] |
| 1B | 1-2 | V608 | Woody plants | USA | V608I | Collado-Romero et al. [[8](#_ENREF_8)] |
| 1B | 1-2 | V609 | Woody plants | USA | V609I | Collado-Romero et al. [[8](#_ENREF_8)] |
| 2A | 1-1 | 395 | Tomato | USA | 395 | Joaquim, 1989 [[2](#_ENREF_2)] |
| 2A | 1-2 | 492 | Tomato | USA | 492 | Joaquim, 1989 [[2](#_ENREF_2)] |
| 2A | 1-2 | V1005 | Olive | Spain | VOR22I | Jiménez-Díaz et al. [[4](#_ENREF_4)] |
| 2A | 1-2 | V1287 | Olive | Spain | VOR855I | Jiménez-Díaz et al. [[4](#_ENREF_4)] |
| 2A | 1-2 | V176 | Cotton | Spain | (X) V176I | Pérez-Artés et al. [[5](#_ENREF_5)], Korolev et al. [[6](#_ENREF_6)], Korolev et al. [[7](#_ENREF_7)], Collado-Romero et al. [[8](#_ENREF_8),[9](#_ENREF_9)] |
| 2A | 1-2 | V213 | Cotton | Spain | V213I | Pérez-Artés et al. [[5](#_ENREF_5)], Korolev et al. 2001, Collado-Romero et al. [[8](#_ENREF_8),[9](#_ENREF_9)] |
| 2A | 1-2 | V404 | Artichoke | Spain | (X) V404II and I | Collado-Romero et al. [[8](#_ENREF_8),[9](#_ENREF_9)] |
| 2A | 1-2 | V496 | Eggplant | Israel | (X) V496I | Collado-Romero et al. [[9](#_ENREF_9)] |
| 2A | 1-2 | V498 | Eggplant | Israel | (X) V498I | Collado-Romero et al. [[9](#_ENREF_9)] |
| 2A | 1-2 | V548 | Artichoke | Spain | V548I | Collado-Romero et al. [[8](#_ENREF_8)] |
| 2B^334^ | 1-2 | V407_II | Artichoke | Spain | V407II | Collado-Romero et al. [[8](#_ENREF_8)] |
| 2B^334^ | 1-2 | V476_I | Artichoke | Spain | V476I | Collado-Romero et al. [[8](#_ENREF_8)] |
| 2B^334^ | 1-2 | V539_I | Artichoke | Spain | V539I | Collado-Romero et al. [[8](#_ENREF_8)] |
| 2B^334^ | 1-2 | V549_I | Artichoke | Spain | V549I | Collado-Romero et al. [[8](#_ENREF_8)] |
| 2B^334^ | 1-2 | V574_I | Artichoke | Spain | V574I | Collado-Romero et al. [[8](#_ENREF_8)] |
| 2B^334^ | 1-2 | V594_I | Artichoke | Spain | V594I | Collado-Romero et al. [[8](#_ENREF_8)], Collado-Romero et al. [[9](#_ENREF_9)] |
| 2B^334^ | 1-2 | V613 | Artichoke | Spain | V613I | Collado-Romero et al. [[8](#_ENREF_8)], Collado-Romero et al. [[9](#_ENREF_9)] |
| 2B^334^ | 1-2 | V675_I | Artichoke | Spain | V675I | Collado-Romero et al. [[8](#_ENREF_8)] |
| 2B^334^ | 1-2 | V679_I | Artichoke | Spain | V679I | Collado-Romero et al. [[8](#_ENREF_8)] |
| 2B^334^ | 1-2 | V681_I | Artichoke | Spain | V681I | Collado-Romero et al. [[8](#_ENREF_8)] |
| 2B^334^ | 1-2 | V685_I | Artichoke | Spain | V685I | Collado-Romero et al. [[8](#_ENREF_8)] |
| 2B^334^ | 1-2 | V695_I | Artichoke | Spain | V695I | Collado-Romero et al. [[8](#_ENREF_8)] |
| 2B^334^ | 1-2 | V700_I | Artichoke | Spain | V700I | Collado-Romero et al. [[8](#_ENREF_8)], Collado-Romero et al. [[9](#_ENREF_9)] |
| 2B^334^ | 1-2 | V702 | Artichoke | Spain | V702I | Collado-Romero et al. [[8](#_ENREF_8),[9](#_ENREF_9)] |
| 2B^R1^ | 1-2 | V1236 | Olive | Spain | VOR348I | Jiménez-Díaz et al. [[4](#_ENREF_4)] |
| 2B^R1^ | 1-2 | V1374 | Olive | Spain | VOR817I | Jiménez-Díaz et al. [[4](#_ENREF_4)] |
| 2B^824^ | 1-2 | V269_I | Cotton | Israel | cot17, V269I | Korolev et al. [[6](#_ENREF_6)], Collado-Romero et al. [[8](#_ENREF_8)] |
| 2B^824^ | 1-2 | V302 | Cotton | Israel | cot117, V302I | Korolev et al. [[6](#_ENREF_6)], Korolev et al. [[7](#_ENREF_7)], Collado-Romero et al. [[8](#_ENREF_8),[9](#_ENREF_9)] |
| 2B^824^ | 1-2 | V408_I | Almond tree | Spain | V408I | Collado-Romero et al. [[9](#_ENREF_9)] |
| 2B^824^ | 1-2 | V474_I | Artichoke | Spain | V474I | Collado-Romero et al. [[8](#_ENREF_8)] |
| 2B^824^ | 1-2 | V481_I | Artichoke | Spain | V481I | Collado-Romero et al. [[8](#_ENREF_8)] |
| 2B^824^ | 1-2 | V534_I | Artichoke | Spain | V534I | Collado-Romero et al. [[8](#_ENREF_8)] |
| 2B^824^ | 1-2 | V546_I | Artichoke | Spain | V546I | Collado-Romero et al. [[8](#_ENREF_8)] |
| 2B^824^ | 1-2 | V639_I | Cotton | Greece | V639I | Collado-Romero et al. [[8](#_ENREF_8)] |
| 2B^824^ | 1-2 | V645_I | Cotton | Greece | V645I | Collado-Romero et al. [[8](#_ENREF_8)] |
| 2B^824^ | 1-2 | V653 | Cotton | Greece | V653I | Collado-Romero et al. [[8](#_ENREF_8)] |
| 2B^824^ | 1-2 | V668_I | Cotton | Turkey | V668I | Collado-Romero et al. [[8](#_ENREF_8)] |
| 2B^824^ | 1-2 | V682_I | Artichoke | Spain | V682I | Collado-Romero et al. [[8](#_ENREF_8)] |
| 2B^824^ | 1-2 | V697 | Artichoke | Spain | V697I | Collado-Romero et al. [[8](#_ENREF_8)] |
| 4A/1A | 1-2 | 131 | Potato | USA | 131_M | Collado-Romero et al. [[9](#_ENREF_9)] |
| 4A | 1-2 | 320 | Potato | USA | 320 | Joaquim, 1989 [[2](#_ENREF_2)] |
| 4A | 1-2 | 21_18 | Potato cv. ‘Snowden’ | USA | 21_18 | Dobinson et al. [[12](#_ENREF_12)], Omer et al. [[13](#_ENREF_13)] |
| 4A | 1-2 | 30_6 | Potato cv. ‘Snowden’ | USA | 30_6 | Dobinson et al. [[12](#_ENREF_12)] |
| 4A | 1-2 | 66_12 | Potato cv. ‘Andover’ | USA | 66_12 | Dobinson et al. [[12](#_ENREF_12)], Omer et al. [[13](#_ENREF_13)] |
| 4A | 1-2 | 83_1 | Potato cv. ‘Burbank’ | USA | 83_1 | Dobinson et al. 2000 |
| 4A | 1-2 | S-228 | Soil | USA | S_228 | Joaquim & Rowe [[3](#_ENREF_3)] |
| 4A | 1-2 | S-55 | Soil | USA | S_55 | Joaquim & Rowe [[3](#_ENREF_3)] |
| 4A | 1-2 | V27 | Potato | USA | V_27 | Joaquim, 1989 [[2](#_ENREF_2)] |
| 4A | 1-2 | VA_102 | Potato | USA | VA_102 | Joaquim, 1989 [[2](#_ENREF_2)] |
| 4A | 1-2 | W_108 | Potato cv. ‘Norkotah’ | USA | W108 | Dobinson et al. [[12](#_ENREF_12)], Omer et al. [[13](#_ENREF_13)] |
| 4A | 1-2 | W-83 | Potato tuber | USA | W83 | Omer et al. [[13](#_ENREF_13)] |
| 4A | 1-2 | W87 | Potato cv. ‘Burbank’ | USA | W87 | Dobinson et al. [[12](#_ENREF_12)], Omer et al. [[13](#_ENREF_13)] |
| 4B | 1-2 | 99_1 | Potato cv. ‘Sebago’ | USA | 99_1 | Dobinson et al. [[12](#_ENREF_12)], Omer et al. [[13](#_ENREF_13)] |
| 4B | 1-2 | S-39 | Soil | USA | S_39 | Joaquim & Rowe [[3](#_ENREF_3)], Botseas & Rowe, [[14](#_ENREF_14)] |
| 4B | 1-2 | S-54 | Soil | USA | S_54 | Joaquim & Rowe [[3](#_ENREF_3)] |
| 4B | 1-2 | V1242 | Olive | Spain | VOR354I | Jiménez-Díaz et al. [[4](#_ENREF_4)] |
| 4B | 1-2 | V160 | Cotton | Israel | cot24, V160 | Korolev et al. [[6](#_ENREF_6)], Korolev et al. [[7](#_ENREF_7)], Collado-Romero et al. [[8](#_ENREF_8),[9](#_ENREF_9)] |
| 4B | 1-2 | V239 | Cotton | Spain | V239I | Korolev et al. [[6](#_ENREF_6)], Collado-Romero et al. [[8](#_ENREF_8)] |
| 4B | 1-2 | WS-4 | Potato | USA | WS_4 | Joaquim, 1989 [[2](#_ENREF_2)] |
| 6 | 1-1 | V560 | Pepper | USA | V560I | Collado-Romero et al. [[8](#_ENREF_8),[9](#_ENREF_9)] |
| 6 | 1-1 | V561 | Pepper | USA | V561I | Collado-Romero et al. [[8](#_ENREF_8),[9](#_ENREF_9)] |

^a^Data are shown for both VCG and lineage only for isolates for which the two are not concordant.

**References**

1. Xu F, Yang L, Zhang J, Guo X, Zhang X, et al. (2012) Prevalence of the defoliating pathotype of *Verticillium dahliae* on cotton in central China and virulence on selected cotton cultivars. J Phytopathol 160: 369-376.

2. Joaquim TR (1989) Vegetative compatibility analysis and comparative pathogenicity to potato of soil and potato plant isolates of *Verticillium dahliae*. PhD Dissertation, Ohio State University PhD.

3. Joaquim TR, Rowe RC (1991) Vegetative compatibility and virulence strains of *Verticillium dahliae* from soil and potato plant. Phytopathology 81: 552-558.

4. Jiménez-Díaz RM, Olivares-García C, Landa BB, Jiménez-Gasco MM, Navas-Cortés JA (2011) Region-wide analysis of genetic diversity in *Verticillium dahliae* populations infecting olive in southern Spain and agricultural factors influencing the distribution and prevalence of vegetative compatibility groups and pathotypes. Phytopathology 101: 304-315.

5. Pérez-Artés E, García-Pedrajas MD, Bejarano-Alcázar J, Jiménez-Díaz RM (2000) Differentiation of cotton-defoliating and nondefoliating pathotypes of *Verticillium dahliae* by RAPD and specific PCR analyses. Eur J Plant Pathol 106: 507-517.

6. Korolev N, Pérez-Artés E, Bejarano-Alcázar J, Rodríguez-Jurado D, Katan J, et al. (2001) Comparative study of genetic diversity and pathogenicity among populations of *Verticillium dahliae* from cotton in Spain and Israel. Eur J Plant Pathol 107: 443-456.

7. Korolev N, Pérez-Artés E, Mercado-Blanco J, Bejarano-Alcázar J, Rodríguez-Jurado D, et al. (2008) Vegetative compatibility of cotton-defoliating *Verticillium dahliae* in Israel and its pathogenicity to various crop plants. Eur J Plant Pathol 122: 603-617.

8. Collado-Romero M, Mercado-Blanco J, Olivares-García C, Valverde-Corredor A, Jiménez-Díaz RM (2006) Molecular variability within and among *Verticillium dahliae* vegetative compatibility groups determined by fluorescent amplified fragment length polymorphism and polymerase chain reaction markers. Phytopathology 96: 485-495.

9. Collado-Romero M, Mercado-Blanco J, Olivares-García C, Jiménez-Díaz RM (2008) Phylogenetic analysis of *Verticillium dahliae* vegetative compatibility groups. Phytopathology 98: 1019-1028.

10. Dervis S, Erten L, Soylu S, Tok FM, Kurt S, et al. (2007) Vegetative compatibility groups in *Verticillium dahliae* isolates from olive in western Turkey. Eur J Plant Pathol 119: 437-447.

11. Dervis S, Mercado-Blanco J, Erten L, Valverde-Corredor A, Pérez-Artés E (2010) Verticillium wilt of olive in Turkey: a survey on disease importance, pathogen diversity and susceptibility of relevant olive cultivars. Eur J Plant Pathol 127: 287-301.

12. Dobinson KF, Harrington MA, Omer M, Rowe RC (2000) Molecular characterization of vegetative compatibility group 4A and 4B isolates of *Verticillium dahliae* associated with potato early dying. Plant Dis 84: 1241-1245.

13. Omer MA, Johnson DA, Rowe RC (2000) Recovery of *Verticillium dahliae* from North American certified seed potatoes and characterization of strains by vegetative compatibility and aggressiveness. Am J Potato Res 77: 1-7.

14. Botseas DD, Rowe RC (1994) Development of potato early dying in response to infection by two pathotypes of *Verticillium dahliae* and co-infection by *Pratylenchus penetrans*. Phytopathology 84: 275-282.
