# Supplementary material for: Recombination between Clonal Lineages of the Asexual Fungus Verticillium dahliae Detected by Genotyping by Sequencing
Source: PLoS One. 2014 Sep 2;9(9):e106740. doi: 10.1371/journal.pone.0106740 (PMC4152335; doi:10.1371/journal.pone.0106740)
Supplement: Table S4 — Number of SNPs and recombination events per contig. Number of SNPs and recombination events for each supercontig relative to the Verticillium dahliae reference genome for isolate VdLs.17. (DOCX) [file pone.0106740.s004.docx]

Table S4. Number of SNPs and recombination events for each supercontig relative to the *Verticillium dahliae* reference genome for isolate VdLs.17 [[1](#_ENREF_1)].

| Chromosome | Supercontig | Supercontig length (nt)^a^ | no. SNPs | no. recombination events |
| --- | --- | --- | --- | --- |
| 1 | 2 | 2529034 | 2446 | 32 |
|  | 7 | 1482790 | 1234 | 21 |
|  | 22 | 624446 | 577 | 7 |
|  | 29 | 371638 | 319 | 4 |
|  | 31 | 235805 | 191 | 2 |
|  | total | 5243713 | 4767 | 66 (66)^b^ |
|  |  |  |  |  |
| 2 | 1 | 2667998 | 2049 | 30 |
|  | 10 | 1129899 | 862 | 13 |
|  | 18 | 824884 | 682 | 6 |
|  | 21 | 641803 | 312 | 6 |
|  | total | 5264584 | 3905 | 55 (55) |
|  |  |  |  |  |
| 3 | 4 | 2001273 | 1456 | 16 |
|  | 6 | 1747785 | 1752 | 32 |
|  | 8 | 1352351 | 974 | 20 |
|  | 27 | 433381 | 405 | 5 |
|  | total | 5534790 | 4587 | 73 (82) |
|  |  |  |  |  |
| 4 | 9 | 1273651 | 417 | 5 |
|  | 11 | 1106702 | 812 | 17 |
|  | 23 | 557237 | 209 | 5 |
|  | 25 | 532607 | 430 | 2 |
|  | 30 | 284126 | 106 | 1 |
|  | total | 3754323 | 1974 | 30 (33) |
|  |  |  |  |  |
| 5 | 15 | 938973 | 753 | 14 |
|  | 16 | 907127 | 889 | 14 |
|  | 17 | 843700 | 755 | 12 |
|  | 24 | 559547 | 394 | 7 |
|  | total | 3249347 | 2791 | 47 (54) |
|  |  |  |  |  |
| 6 | 5 | 1801442 | 1438 | 19 |
|  | 19 | 736048 | 531 | 5 |
|  | total | 2537490 | 1969 | 24 (30) |
|  |  |  |  |  |
| 7 | 3 | 2207176 | 2052 | 46 |
|  | 26 | 499255 | 558 | 10 |
|  | 28 | 441290 | 331 | 4 |
|  | total | 3147721 | 2941 | 60 (54) |
|  |  |  |  |  |
| 8 | 12 | 1018472 | 742 | 13 |
|  | 13 | 1020271 | 748 | 14 |
|  | 14 | 946171 | 779 | 13 |
|  | total | 2984914 | 2269 | 40 (37) |
|  |  |  |  |  |
| Supercontigs not assigned to chromosomes | 20 | 643454 | 618 | 13 |
|  | 32 | 212308 | 279 | 5 |
|  | 33 | 162476 | 81 | 0 |
|  | 34 | 149246 | 136 | 1 |
|  | 35 | 127187 | 27 | 0 |
|  | 36 | 133489 | 91 | 2 |
|  | 37 | 135517 | 88 | 0 |
|  | 38 | 108517 | 62 | 0 |
|  | 39 | 99863 | 35 | 0 |
|  | 40 | 90006 | 22 | 0 |
|  | 41 | 63491 | 60 | 0 |
|  | 42 | 62837 | 24 | 0 |
|  | 43 | 25257 | 4 | NA^c^ |
|  | 44 | 20440 | 8 | 0 |
|  | 45 | 15454 | 7 | 0 |
|  | 46 | 11865 | 0 | NA |
|  | 47 | 11719 | 0 | NA |
|  | 48 | 11324 | 0 | NA |
|  | 49 | 9865 | 2 | 0 |
|  | 50 | 9223 | 1 | NA |
|  | 51 | 3843 | 0 | NA |
|  | 52 | 4190 | 0 | NA |

^a^ contig size determined by Klosterman et al. [[1](#_ENREF_1)]

^b^ number of recombination events detected for contigs concatenated on each chromosome are shown in parentheses

^c^ Not applicable because not enough SNPs were genotyped to detect recombination

**Reference**

1. Klosterman SJ, Subbarao KV, Kang S, Veronese P, Gold SE, et al. (2011) Comparative genomics yields insights into niche adaptation of plant vascular wilt pathogens. PLoS Path 7: e1002137.
